# Efficacy and safety of 6% hydroxyethyl starch 130/0.4 (Voluven) for perioperative volume replacement in children undergoing cardiac surgery: a propensity-matched analysis

**DOI:** 10.1186/s13054-015-0830-z

**Published:** 2015-03-17

**Authors:** Philippe Van der Linden, Melanie Dumoulin, Celine Van Lerberghe, Cristel Sanchez Torres, Ariane Willems, David Faraoni

**Affiliations:** Department of Anesthesiology, University Hospital Brugmann and Queen Fabiola Children’s University Hospital, Free University of Brussels, 4 Place Van Gehuchten, B-1020 Brussels, Belgium; Pediatric Intensive Care Unit, Queen Fabiola Children’s University Hospital, Free University of Brussels, 15 Avenue JJ Crocq, B-1020 Brussels, Belgium; Department of Anesthesiology, Perioperative and Pain Medicine, Boston Children’s Hospital, Harvard Medical School, 300 Longwood Avenue, Boston, MA 02115 USA

## Abstract

**Introduction:**

Six percent hydroxyethyl starch (HES) 130/0.4 is considered an alternative to human albumin (HA) and crystalloids for volume replacement in children undergoing cardiac surgery. In this large propensity-matched analysis, we aimed to assess the efficacy and safety of replacing HA with HES for intraoperative volume therapy in children undergoing cardiac surgery with cardiopulmonary bypass (CPB).

**Methods:**

We retrospectively reviewed our database, including children who underwent cardiac surgery between January 2002 and December 2010. Four percent HA was used until 2005; it was replaced by HES thereafter. Demographic data, intra- and postoperative blood loss and blood component transfusions were recorded, together with the incidence of postoperative complications and mortality. We performed a propensity-matched analysis using 13 possible confounding factors to compare children who received either HES or HA intraoperatively. The primary objectives included the effects of both fluids on intraoperative fluid balance (difference between fluids in and fluids out (efficacy)) and blood loss and exposure to allogeneic blood products (safety). Secondary safety outcomes were mortality and the incidence of postoperative renal dysfunction.

**Results:**

Of 1,832 children reviewed, 1,495 were included in the analysis. Intraoperative use of HES was associated with a less positive fluid balance. Perioperative blood loss, volume of red blood cells and fresh frozen plasma administered, as well as the number of children who received transfusions, were also significantly lower in the HES group. No difference was observed regarding the incidence of postoperative renal failure requiring renal replacement therapy or of morbidity and mortality.

**Conclusions:**

These results confirm that the use of HES for volume replacement in children during cardiac surgery with CPB is as safe as HA. In addition, its use might be associated with less fluid accumulation. Further large studies are needed to assess whether the reduction in fluid accumulation could have a significant impact on postoperative morbidity and mortality.

## Introduction

Maintenance of normovolemia remains a major challenge during cardiac surgery with cardiopulmonary bypass (CPB). It is now accepted that both hypovolemia and fluid overload are associated with increased morbidity and mortality [1,2]. During cardiac surgery, a relatively large amount of fluid is administered to optimize cardiac output in the context of a drug-induced vasodilation and to compensate for surgical blood loss [3]. In addition, the use of acellular fluids to prime the CPB results in acute hemodilution and significantly contributes to the positive fluid balance achieved at the end of the surgery [4]. Management of hemodilution is particularly challenging in the pediatric cardiac population, owing to the higher ratio between the priming volume and the children’s circulating blood volume [5].

Human albumin (HA) and crystalloids remain first choices for CPB priming and volume replacement in the perioperative period of pediatric cardiac surgery [6]. Compared with crystalloids, the administration of HA in the CPB prime decreased the intraoperative positive balance [7]. Although HA allows for the maintenance of an adequate oncotic pressure [8,9], its cost remains high, which leads physicians to look for less expensive alternatives. Third-generation hydroxyethyl starches (HES; for example, tetrastarches) have been developed. They appear to have interesting pharmacokinetic properties and are five times cheaper than HA. As a result of a quicker achieved optimal *in vivo* molecular weight, 6% HES 130/0.4 offers fluid volume expansion comparable to that of older HES, whereas its effects on hemostasis appear to be less marked [10]. Recently, the safety of 6% HES 130/0.4 has been questioned in adult patients who are critically ill. In a prospective, randomized, double-blind study including about 7,000 patients, administration of 6% HES 130/0.4 was associated with an increased need for renal replacement therapy compared with use of isotonic saline [11]. In another randomized double-blind trial, which included 804 patients with severe sepsis, the use of balanced HES 130/0.4 was associated with increased 90-day mortality and an increased need for renal replacement therapy compared with Ringer’s acetate [12]. Although the results of these studies led to an intense, and sometimes emotional, debate, they should be interpreted with caution, taking into account the clinical context [13], and cannot be transposed to the pediatric cardiac population.

Only a few studies have assessed the efficacy and safety of 6% HES 130/0.4 in the pediatric population. In 2009, 6% HES 130/0.4 was compared with 4% HA for perioperative volume replacement therapy in one blinded, single-center, randomized trial that included 119 children undergoing cardiac surgery with CPB [14]. In that study, 6% HES 130/0.4 was associated with comparable perioperative blood loss, but with a lower intraoperative fluid balance, compared with 4% HA. These results were confirmed in a two-center, double-blind, prospective study where 6% HES 130/0.4 was compared with 5% HA in the same population (n = 61) [15]. However, neither study was sufficiently powered to provide any firm conclusion regarding the safety of 6% HES 130/0.4 in this population.

In the present large, retrospective, propensity-matched study, we assessed the efficacy and safety of replacing HA with 6% HES 130/0.4 for volume replacement therapy in children undergoing cardiac surgery with CPB at our department. Our hypothesis was that HES 130/0.4 is not inferior to HA regarding our primary and secondary objectives. The primary objective for efficacy was to assess the relationship between administration of 6% HES 130/0.4 and intraoperative fluid balance, and the primary safety objective was to assess its relationship with blood loss and exposure to allogeneic blood products. As secondary objectives, we assessed the effect of 6% HES 130/0.4 on the incidence of postoperative morbidity, including the incidence of renal failure requiring renal replacement therapy and mortality.

## Materials and methods

After receiving approval from the Queen Fabiola Children’s University Hospital ethics committee (CEH10/13), we retrospectively reviewed our departmental database that included all children who underwent cardiac surgery with CPB between January 2002 and December 2010. Children in a moribund state (American Society of Anesthesiologists (ASA) physical status V), Jehovah’s witnesses and those with missing data were excluded. We also excluded children younger than 1 month of age because these patients received primarily fresh frozen plasma (FFP) in the CPB prime and no colloid was administered. The local ethics board waived the requirement for written informed consent because of the retrospective nature of the protocol.

During the study period, children were treated by the same team, including two experienced surgeons, three experienced anesthesiologists and two experienced intensive care unit (ICU) pediatricians. In the operating room, the anesthetic technique remained globally unchanged. Monitoring included pulse oximetry, five-lead electrocardiography, non-invasive arterial pressure, arterial and central venous pressures, urinary output and cutaneous and rectal temperature probes. Intravenous anesthesia based on midazolam, sufentanil and rocuronium was preferred in all children, with the exception of children with univentricular physiology, who underwent a cavopulmonary connection and in whom anesthesia was performed with propofol or sevoflurane, remifentanil and atracurium. All children received cefazolin 25 mg∙kg^−1^ and methylprednisolone 30 mg∙kg^−1^ after the induction of anesthesia. Antifibrinolytic agents were routinely used in our department. Aprotinin was used before 2008; it was replaced thereafter by tranexamic acid. Before aortic cannulation, 4 mg∙kg^−1^ unfractionated heparin (UFH) was administered to reach an activated clotting time (ACT) longer than 480 seconds. Anticoagulation level was regularly checked during CPB using repeated ACT measures (ACT II monitor; Medtronic, Kerkrade, the Netherlands), and additional UFH boluses were given to maintain ACT longer than 480 seconds during the whole CPB operation. At the end of CPB, protamine was administered (dose: half of the total UFH dose administrated during the whole CPB procedure) to antagonize heparin activity. Adequate reversal was controlled using the ACT II monitor comparing ACT measured in cartridges with and without heparinase (Medtronic).

The CPB circuit was primed primarily with 4% HA between 2002 and 2005 and with 6% HES 130/0.4 in 0.9% sodium chloride (Voluven; Fresenius Kabi, Bad Homburg, Germany) after this period. In addition, 20% mannitol (1.5 ml∙kg^−1^), sodium bicarbonate (20 mEq∙L^−1^) and UFH (50 mg∙L^−1^) were added to the prime. Different models of oxygenator chosen on the basis of the child’s body weight were used during the study period. In addition, new miniaturized oxygenators, which require a smaller prime volume, were progressively introduced in our department starting in 2008.

When preparing the CPB prime, the hematocrit level to be achieved on bypass was calculated based on the volume of the prime and the estimated blood volume (EBV) of the child. Packed red blood cells (RBCs) were added in the prime when the predicted hematocrit after cardioplegia (crystalloid cold balanced solution enriched with potassium chloride 30 mmol∙L^−1^) was estimated to fall below 20%. During CPB, body temperature was decreased according to the length of aortic clamp duration and the complexity of the surgery. The body temperature of all patients was rewarmed above 35.5°C before weaning from CPB. After weaning, modified ultrafiltration (MUF) was used to increase hematocrit of the residual blood volume in the circuit.

For intraoperative volume replacement, including CPB priming, the patients could receive up to 50 ml/kg/day of either 6% HES 130/0.4 or HA. For intraoperative volume replacement before or after the CPB, the amount of the colloid not used for priming could be given, up to the maximum dosage for the individual patient, if needed. No specific algorithm for fluid administration was used. Infusion rates were adjusted to individual needs at the discretion of the anesthesiologist in charge of the patient, to maintain a mean arterial pressure within the range of 50 to 85 mmHg. If the maximum dose of 6% HES 130/0.4 was reached, HA was used as a rescue colloid. The use of inotropes and vasopressors was left to the discretion of the anesthesiologist, and no specific algorithm was applied.

Our RBC transfusion policy was standardized in agreement with the Department of Anesthesiology and the Pediatric Intensive Care Unit (PICU). We adopted a restrictive transfusion strategy during the study period, and this policy was maintained the same for the entire operative period and PICU stay for every patient included in this study. After each patient was separated from CPB, RBCs were transfused to maintain a hematocrit level above 24% in cases of abnormal bleeding or to increase oxygen delivery in cases of persistent lactic acidosis after optimization of cardiac output with inotropes, vasoactive agents or both. In case of abnormal bleeding, defined as a diffuse bleeding in the surgical field that could not be controlled by packing sponges and/or application of topic hemostatic agents after adequate heparin antagonization with protamine, FFP was administered at the dose of 15 ml∙kg^−1^. The same dose was repeated in case of persistent bleeding. In addition, platelets were administered in cases of significant blood loss associated with a platelet count less than 100 × 10^3^/μl, as measured by using our standard laboratory tests.

Recorded data included age (months), preoperative weight (kg), height (cm), preoperative oxygen saturation (%), the presence of a cyanotic disease (defined as preoperative oxygen saturation less than 90% by pulse oximetry) and ASA physical status and Risk Adjustment for Congenital Heart Surgery (RACHS-1) score. The RACHS-1 score was used to define the complexity of the surgical procedure [16]. It uses six categories of surgical risk, with a score of 1 representing the lower risk and 6 the highest. The incidence of preoperative cardiac failure and previous cardiac surgery with or without sternotomy was also recorded. Intraoperative characteristics, including duration of surgery, CPB and aortic cross-clamp time and minimal body temperature while on CPB were recorded. The degree of hemodilution was measured in milliliters per kilogram using the ratio between the CPB prime volume in milliliters per kilogram and the child’s EBV in milliliters per kilogram. The use of MUF and the amount of MUF in milliliters per kilogram were also recorded. The total fluid volume administered intraoperatively included the CPB prime volume, the cardioplegia volume and all fluid administered with drugs and flushes of invasive pressure lines. The total output included blood loss, urine output and the amount of ultrafiltration. No cell salvage device was used during the study period. Weighting sponges and measured surgical suction were used to determine intraoperative blood loss, considering that irrigation volume was measured and separated from the main surgical suction. In the postoperative period, measured chest tube drainage was used to assess blood loss. In addition, we calculated blood loss according to the following formula, adapted a previously publication [17]:

$$ \mathrm{Calculated}\ \mathrm{blood}\ \mathrm{loss}\ \left(\mathrm{ml}.{\mathrm{kg}}^{-1}\right) = \frac{\Big(\left(\mathrm{E}\mathrm{B}\mathrm{V} \times \mathrm{H}\mathrm{c}\mathrm{t}\ \mathrm{preop}\right)\ \hbox{--}\ \left(\mathrm{E}\mathrm{B}\mathrm{V} \times \mathrm{H}\mathrm{c}\mathrm{t}\ \mathrm{POD}3\right) + \left(\mathrm{RBCs}\ \mathrm{t}\mathrm{ransfused}\ \mathrm{up}\ \mathrm{t}\mathrm{o}\ \mathrm{POD}3\ \left(\mathrm{ml}\right) \times 0.7\right)}{\mathrm{Patient}\ \mathrm{body}\ \mathrm{weight}}, $$

where EBV is estimated blood volume in milliliters, Hct is hematocrit expressed in percent, POD3 is postoperative day 3, 0.7 is mean hematocrit of the RBC units and patient body weight is preoperative body weight in kilograms.

The incidence of RBCs, FFP and platelet concentrates transfused intraoperatively and during the first 3 postoperative days was recorded. Hemoglobin level in grams per liter, hematocrit in percent and creatinine level in milligrams per deciliter were systematically measured in the immediate preoperative period and on postoperative days 1 and 3. Postoperative outcome data recorded included the incidence of surgical reexploration for bleeding, duration of mechanical ventilation, incidence of infection, neurological complications (for example, postoperative apparition of a neurological deficit, coma, seizures), renal replacement therapy and in-hospital mortality.

### Statistical analysis

An independent statistician blinded to the type of colloid used performed the whole statistical analysis. Descriptive statistics were performed for each variable recorded in our database.

We defined *a priori* 13 confounding variables to be used in the propensity-matched analysis for children included in the 2 groups: age, sex, preoperative weight, height, ASA physical status, the presence of a cyanotic disease, history of previous cardiac surgery, history of cardiac failure, preoperative hemostatic disorder (defined as platelet count less than 100 × 10^3^/μl, fibrinogen level less than 100 mg∙dl^−1^, prothrombin time and activated partial thromboplastin time 1.5 times above upper limit of normal), scheduled surgery, RACHS-1 score, EBV and the administration of antifibrinolytic agents. We used genetic matching, a generalization of propensity score and Mahalanobis distance that maximizes the balance of observed covariates between treated and control groups [18]. The algorithm uses a genetic algorithm to optimize balance as much as possible, given the data. The method is nonparametric and does not depend on knowing or estimating the propensity score. The genetic matching attempts to minimize a measure of the maximum observed discrepancy between the matched treated and control covariates at every iteration of optimization. The algorithm attempts to minimize the largest observed covariate discrepancy at every step, and this is accomplished by maximizing the smallest *P*-value at each step. The algorithm stopped when the difference between the last four solutions was small. We performed a one-to-one genetic matching with replacement. Last, an absolute standardized difference less than 10% was considered to support the assumption of balance between the groups because it is not affected by the sample size, unlike *P*-values, and it may be used to compare the relative balance of variables measured in different units [19,20]. The importance of assessing the balance of baseline covariates in the matched sample was done using standardized differences and empirical quantile-quantile statistics. Because matched samples are no longer independent, bootstrap Kolmogorov–Smirnov tests and paired *t*-tests were calculated [21,22]. The mean and standard deviation obtained after matching are presented for continuous variables, and the percentage is presented for categorical variables.

After matching, we used logistic regression for binary outcome variables and weighted least squares (WLS) for continuous outcome variables, including the treatment group effect, the variables used for the matching score as covariates, hemofiltration and volume priming (excepted for those we observed multicollinearity with the dependent variable: hemofiltration, volume priming, ICU infection, ICU length of stay) and the weight resulting from the genetic matching. The estimators in the WLS were weighted for one per fitted value of a first linear model. We applied a Bonferroni correction to the results. Because a total of 19 linear and logistic regressions were performed, *P* <0.00263 (0.05/19) was considered statistically significant. Last, we performed linear mixed models regression to compare evolution of hemoglobin and creatinine between both groups based on multiple imputations of missing values [23].

Statistical analyses were performed with Prism 6 for Mac OS software (version 6.0d; GraphPad Software, La Jolla, CA, USA) and R software version 3.0.1 (R Foundation for Statistical Computing, Vienna, Austria) using the packages ‘Matching’ and ‘rgenoud’ for the match-propensity score subanalysis [18].

## Results

### Demographic data

**Figure 1:**
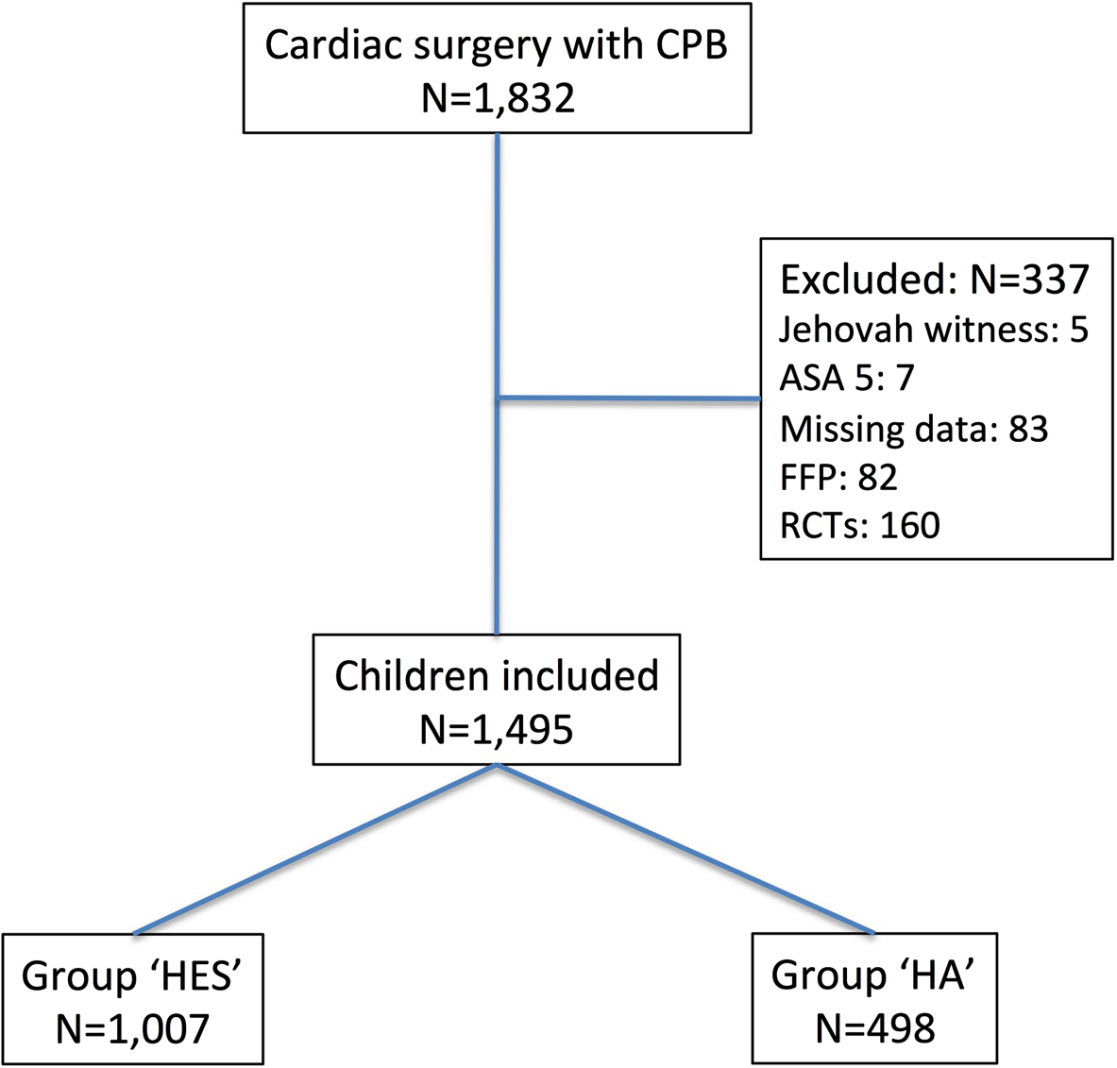
**Flowchart of the study.** ASA, American Society of Anesthesiologists physical status; CPB, Cardiopulmonary bypass; FFP, Fresh frozen plasma; HA, Human albumin; HES, Hydroxyethyl starch; RCT, Randomized controlled trial.

Of the 1,832 children included in our departmental database, 1,495 were included in the final analysis (Figure 1). We excluded 83 children because relevant data were missing, 82 because FFP was used primarily in the CPB prime, 7 who were in a moribund state and 5 Jehovah’s witnesses. In addition, 160 children were voluntarily excluded because they had already participated in 1 of the 2 prospective trials performed in our department, in which we compared 6% HES 130/0.4 with HA [14,15].

Demographic characteristics of the studied population are reported in Table 1. Before matching, children included in the HA group were significantly younger (*P* = 0.049), had a lower preoperative body weight (*P* = 0.009), more frequently had a cyanotic disease (*P* <0.001) and more often had undergone previous surgery (*P* <0.001). After matching, the absolute standardized differences were clearly under 10, suggesting that we may consider the groups as being equal on the selected covariates.

**Table 1:** **Demographic characteristics of children included before and after matching** ^**a**^

		**Before matching**				**After matching**				
**Variable**	**Group HES**	**HA group**	**D**	***P*** **-value**	**ASD**	**HES group**	**HA group**	**D**	***P*** **-value**	**ASD**
	**(n = 1007)**	**(n = 488)**				**(n**_**os**_ **= 1007)**	**(n**_**os**_ **= 322)**			
						**(n**_**m**_ **= 1007)**	**(n**_**m**_ **= 1007)**			
Age (mo)	34.6 (44.1)	28.1 (36.6)	0.036	0.049	14.642	34.6 (44.1)	34.3 (44.4)	0.01	0.501	0.64733
Height (cm)	82.5 (28.1)	78.1 (25.1)	0.075	0.034	15.702	82.5 (28.1)	82.9 (28.3)	0.03	0.663	1.6333
Weight (kg)	11.5 (10.2)	9.7 (8.9)	0.094	0.009	17.825	11.5 (10.1)	11.4 (9.9)	0.036	0.490	0.96439
ASA physical status	3.0 (0.4)	3.2 (0.5)	0.195	<0.001	58.814	3.0 (0.4)	3.0 (0.4)	0.001	0.999	0.2368
Male sex (%)	543 (54)	288 (59)	0.057	0.036	11.447	543 (54)	182 (57)	0.026	0.092	5.1772
Cyanotic disease (%)	433 (43)	254 (52)	0.091	<0.001	18.477	433 (43)	138 (43)	0.005	0.297	1.0027
Redo surgery (%)	164 (16.3)	47 (9.6)	0.066	<0.001	18.014	164 (16.3)	52 (16.1)	0.002	0.655	0.53762
Hemostatic disorder (%)	29 (2.9)	11.2 (2.3)	0.006	0.464	3.7397	29 (2.9)	9 (2.7)	0.002	0.157	1.187
Preoperative cardiac failure (%)	192 (19.1)	96 (19.7)	0.006	0.782	1.5409	192 (19.1)	57 (17.9)	0.011	0.210	2.7794
Elective surgery (%)	996 (99)	473 (97)	0.016	0.038	15.785	996 (99)	319 (99)	0.002	0.317	1.759
RACHS-1 score	2.5 (0.8)	2.6 (0.8)	0.100	0.174	8.8267	2.5 (0.8)	2.5 (0.7)	0.065	0.366	1.9669
Antifibrinolytics (%)	967 (96)	473 (97)	0.003	0.5101	3.4356	967 (96)	309 (96)	0.001	0.083	1.4894
Estimated blood volume (ml)	865 (681)	737 (599)	0.051	0.001	18.755	865 (681)	857 (669)	0.011	0.438	1.2004

^a^Data are expressed as mean and standard deviation or as number and percentage. ASA, American Society of Anesthesiologists; ASD, Absolute standardized difference; D, D-statistic is the maximum difference in the empirical quantile-quantile plot, and it is sensitive to imbalance across the empirical distribution; n_m_, Number of matched observations; n_os_, Number of observations in the original sample; RACHS, Risk Adjustment for Congenital Heart Surgery.

The main comparisons between the two study groups are reported in Table 2. After adjustment for the confounding variables, the weight of matching and the Bonferroni correction (*P*-value significant if below 0.00263), CPB (*P* = 0.001) and surgery duration (*P* = 0.001) were both significantly decreased in the HES group. The priming volume (*P* <0.001) and the degree of hemodilution (*P* <0.001) were also lower in the HES group, whereas the minimal body temperature reached on bypass was significantly lower in the HA group.

**Table 2:** **Comparison between groups for operative characteristics and outcomes after adjustment for confounding variables** ^**a**^

**Variables**	**HES group**	**HA group**	**Adjusted** ***P*** **-value**
	**(n**_**os**_ **= 1007)**	**(n**_**os**_ **= 322)**	
	**(n**_**m**_ **= 1007)**	**(n**_**m**_ **= 1007)**	
Surgery duration (min)	217.4 (67.8)	227.5 (98.5)	0.082
CPB duration (min)	110.8 (45.4)	117.6 (53.8)	0.013
Aortic clamping (%)	904 (89.8)	303 (94.4)	0.100
Minimum temperature during CPB (°C)	30.4 (2.9)	28.4 (3.8)	<0.001^b^
Priming volume (ml∙kg^−1^)	65.7 (34.7)	111.5 (54.7)	<0.001^b^
Degree of hemodilution (%)^c^	80.1 (36.9)	136.4 (58.2)	<0.001^b^
MUF (%)	921 (91.6)	919 (91.4)	0.41
MUF (ml∙kg^−1^)	29.9 (16.5)	31.3 (17.6)	0.86
Exposure to blood products (%)	639 (63.5)	801 (79.5)	<0.001^b^
Reexploration for bleeding (%)	7 (0.7)	6 (0.6)	0.79
ICU infection (%)	438 (43.6)	445 (44.3)	0.57
Neurological disorder (%)	27 (2.7)	37 (3.7)	0.53
Postoperative cardiac assistance (%)	13 (1.3)	20 (2.0)	0.90
Renal replacement therapy (%)	11 (1.1)	14 (1.4)	0.17
ICU length of stay (days)	7.5 (10.0)	7.0 (6.7)	0.41
Length of hospital stay (days)	19.7 (16.6)	20.1 (16.2)	0.10
In-hospital mortality (%)	21 (2.1)	7 (2.3)	0.24

^a^CPB, Cardiopulmonary bypass; HA, Human albumin; HES, Hydroxyethyl starch; ICU: intensive care unit; MUF, Modified ultrafiltration; n_m_, Number of matched observations; n_os_, Number of observations in the original sample. ^b^Variable significantly different between the two groups after applying the Bonferroni correction. ^c^Degree of hemodilution is presented as the mean percentage hemodilution with standard deviation in parentheses.

### Primary efficacy objective

**Figure 2:**
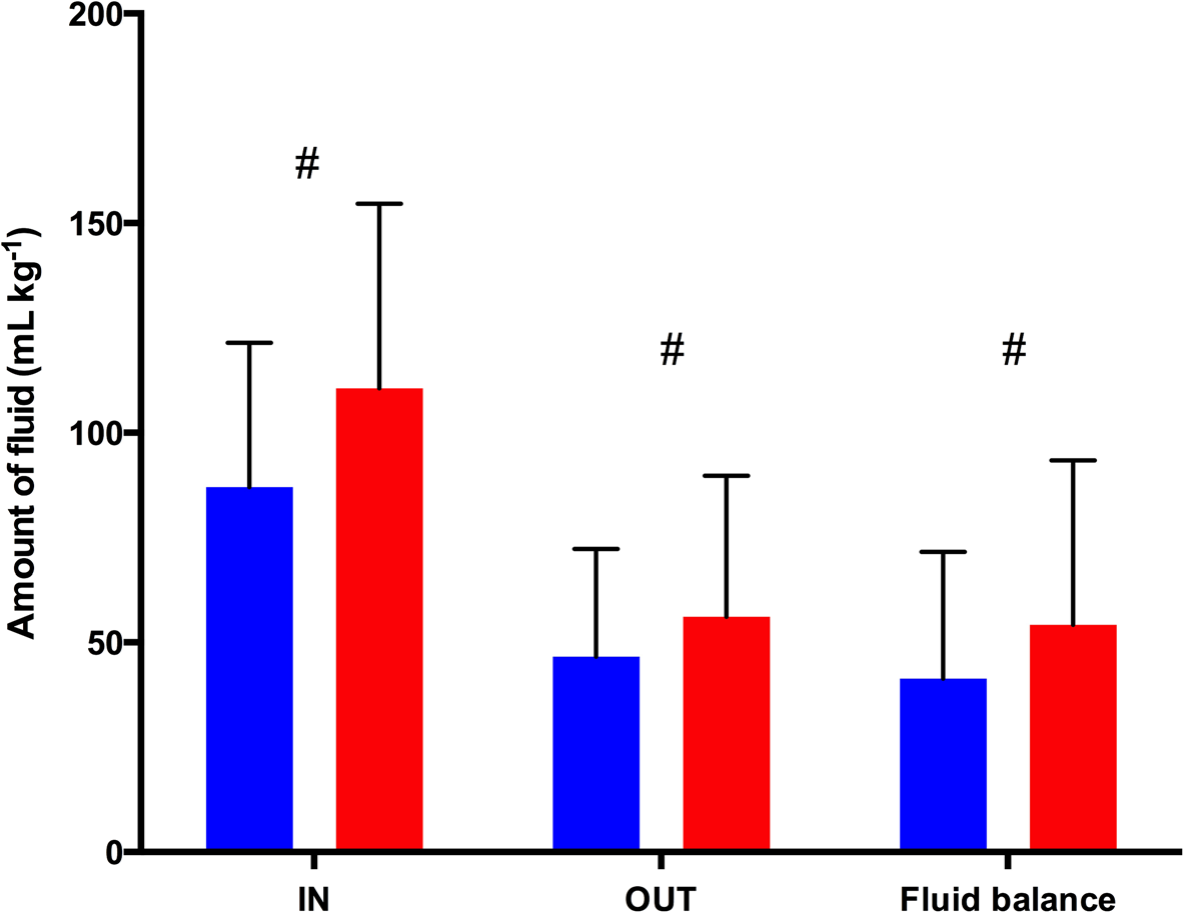
**Difference in fluid balance between groups.** Red bars represent the human albumin group, and blue bars represent the hydroxyethyl starch group. Data are mean ± standard deviation. #*P* <0.001. IN, Total fluid volume administered intraoperatively, including cardiopulmonary bypass prime volume, cardioplegia volume and all fluid administered with drugs and flushes of invasive pressure lines; OUT, Total fluid output, including blood loss, urine output and amount of ultrafiltration.

A significant difference was observed for fluid administration, with higher intake (110.6 ± 44.0 ml∙kg^−1^ for HA group vs. 87.0 ± 44.0 ml∙kg^−1^ for HES group) and output (56.1 ± 33.6 ml∙kg^−1^ for HA group vs. 46.6 ± 25.6 ml∙kg^−1^ for HES group) in children included in the HA group (both *P* <0.001) (Figure 2). However, the fluid balance remained more positive in the HA group (54.1 ± 39.2 ml∙kg^−1^ vs. 41.3 ± 30.2 ml∙kg^−1^; *P* <0.001). MUF was similarly used in both groups, such as the volume of ultrafiltration, which was not significantly higher in the HA group after Bonferroni correction (29.9 ± 16.5 ml∙kg^−1^ vs. 31.3 ± 17.6 ml∙kg^−1^; *P* <0.01).

### Primary safety objective

**Figure 3:**
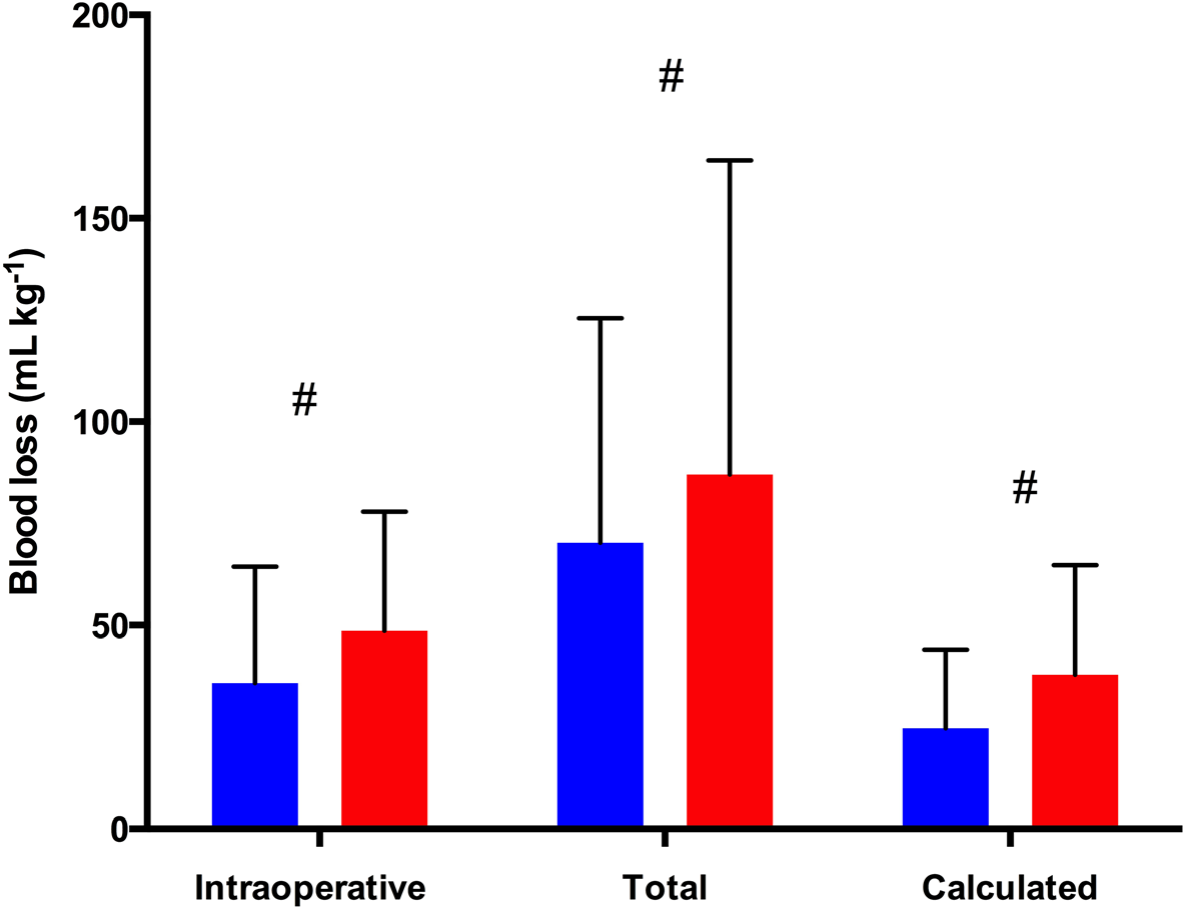
**Comparison of perioperative blood loss between groups.** Total blood loss includes the amount of blood lost intraoperatively and the blood collected in the chest tubes. Calculated blood loss was determined according to a formula adapted from a previous publication [17] (see Materials and methods section). Red bars represent the human albumin group, and blue bars represent the hydroxyethyl starch group. Data are mean ± standard deviation. #*P* <0.001.

**Figure 4:**
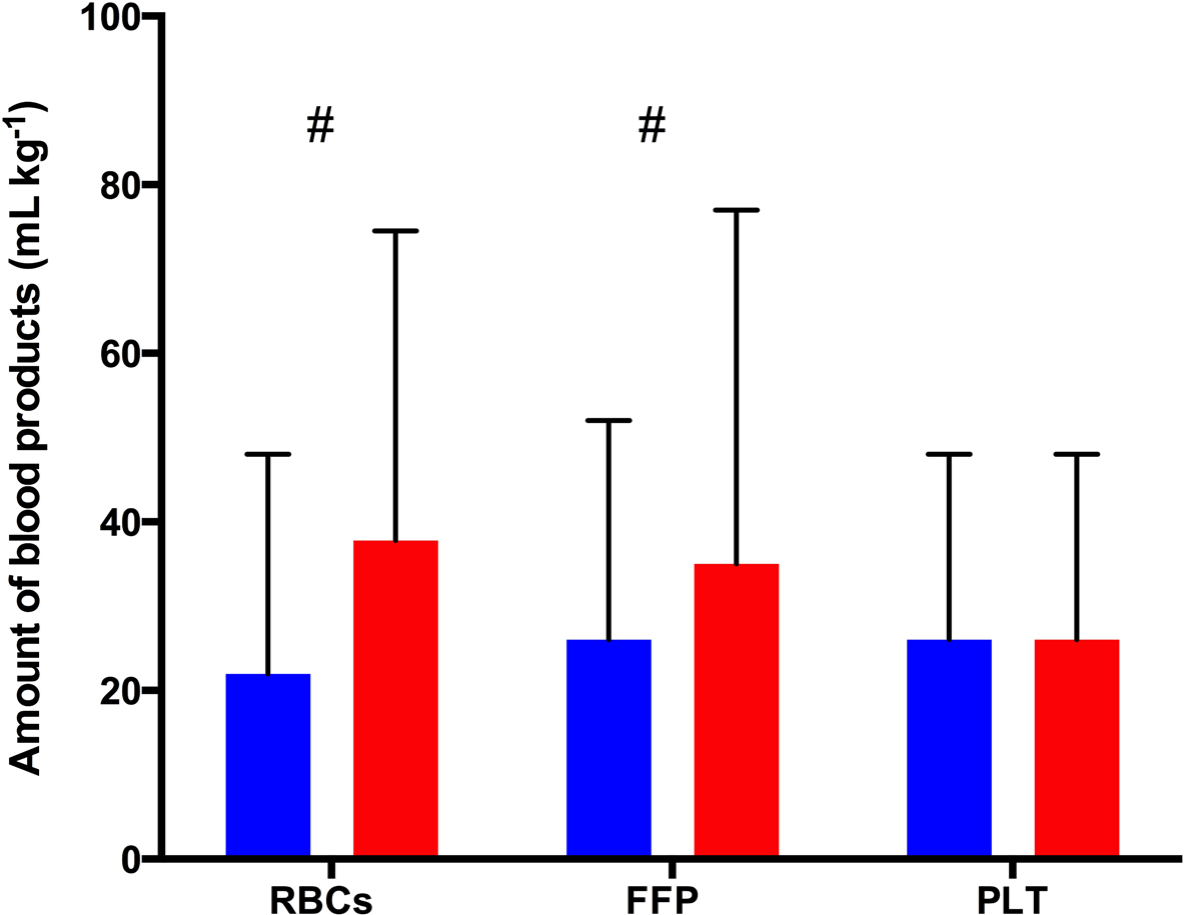
**Difference in amount of blood products transfused between groups intraoperatively and during the first 3 postoperative days.** FFP, Fresh frozen plasma; PLT, Platelet concentrates; RBCs, Packed red blood cells. Red bars represent the human albumin group, and blue bars represent the hydroxyethyl starch group. Data are mean ± standard deviation. #*P* <0.001.

Regarding perioperative bleeding (Figure 3), intraoperative (48.6 ± 28.6 ml∙kg^−1^ for HA group vs. 35.8 ± 28.6 ml∙kg^−1^ for HES group), total (87.1 ± 77.2 ml∙kg^−1^ for HA group vs. 70.3 ± 55.2 ml∙kg^−1^ for HES group) and calculated blood losses (37.9 ± 26.9 ml∙kg^−1^ for HA group vs. 24.7 ± 19.3 ml∙kg^−1^ for HES group) were significantly lower in the HES group (*P* <0.001). Exposure to any blood product intraoperatively and during the first 3 postoperative days was significantly lower in the HES group (Table 2), and this was essentially related to a lower exposure to RBCs (66% vs. 83%; *P* <0.001). The amounts of RBCs and FFP transfused were also significantly lower in the HES group (Figure 4). Interestingly, hemoglobin levels in the preoperative and immediate postoperative periods were not different between the two groups (Table 3).

**Table 3:** **Fixed-effects comparison between groups for hemoglobin and creatinine after adjustment for confounding variables** ^**a**^

	**Groups**	**Preoperative**	**Postoperative day 1**	**Postoperative day 3**	**Adjusted** ***P*** **-value**
Hemoglobin (g∙L^−1^)	HES	135 ± 29	109 ± 19	105 ± 18	0.503
	HA	133 ± 30	110 ± 22	107 ± 20	
Creatinine (mg∙dl^−1^)	HES	0.35 ± 0.17	0.37 ± 0.18	0.28 ± 0.17	<0.001
	HA	0.43 ± 0.26	0.45 ± 0.30	0.41 ± 0.39	

^a^HA, Human albumin; HES, Hydroxyethyl starch.

### Secondary objectives

No difference was observed regarding the incidence of postoperative complications, and the use of HES was not associated with an increased incidence of renal failure or requirement for renal replacement therapy (Table 2). Preoperative creatinine level was lower in the HES group and remained lower in the immediate postoperative period (Table 3). No difference between groups was found regarding the length of ICU stay, length of hospital stay or mortality.

## Discussion

In this large propensity-matched study, 6% HES 130/0.4 represented an effective and safe alternative to HA in children undergoing cardiac surgery with CPB. These results confirm those obtained in two other prospective trials [14,15] in which administration of HES 130/0.4 allowed for a significant reduction in fluid balance without any increase in blood loss, blood product transfusion requirement and side effects. The present study included the largest cardiac pediatric population ever studied with regard to HES treatment.

Regarding our primary efficacy objective, the results of our study indicate that the use of 6% HES 130/0.4 was associated with a significantly lower intraoperative fluid balance. High intraoperative fluid balance has been shown to increase ICU length of stay in adult patients undergoing coronary artery bypass graft surgery [24]. Intraoperative positive fluid balance could also contribute to postoperative fluid overload, which has been shown to significantly affect patient outcome. Hazle *et al*. observed that early postoperative fluid overload after cardiac surgery was associated with bad outcomes in infants under the age of 6 months [5]. They concluded that fluid overload or daily weight gain was well correlated with the incidence of acute kidney injury, which increases postoperative morbidity and mortality. In a recent large study, Hassinger *et al*. confirmed that early postoperative fluid overload preceded acute kidney injury and was associated with higher morbidity in pediatric cardiac surgery patients between 2 weeks and 18 years of age [2]. Seguin *et al*. reported recently that fluid overload occurs early after cardiac surgery in children and is associated with prolonged PICU length of stay and ventilation [25]. In our study, the use of 6% HES 130/0.4 might have been associated with less fluid accumulation in the early postoperative period, as it may have provided a better oncotic pressure than 4% HA, which is slightly hypo-oncotic [26]. However, the results we report cannot be attributed solely to the replacement of HA by HES, as, during the studied period, CPB management was modified to decrease the priming volume and therefore the degree of hemodilution achieved in our pediatric population during CPB. However, our findings are in accord with those of two other prospective randomized studies performed at our institution, in which the use of HES was associated with a reduction in the intraoperative fluid balance while CPB management was maintained unaltered [14,15].

Regarding the primary safety objective, our results indicate that the use of 6% HES 130/0.4 was associated with a reduced exposure to RBC and FFP transfusion in the studied population. These results do not necessarily support a superiority of the tetrastarches to HA, because many other factors, such as the reduction of the degree hemodilution associated with the miniaturization of the CPB circuitry, may have play a role in the reduction of blood component transfusion. However, it should be noted that both measured and calculated perioperative blood losses were significantly lower in the HES group than in the HA group during a period when our restrictive transfusion policy was not modified. These results are again in agreement with those of our previous prospective randomized studies, in which we reported a reduction [14], or a trend [15] toward a reduction, in the exposure to allogeneic blood products. They are also in agreement with those of a recent meta-analysis [27].

Regarding our safety secondary objective, in our large dataset, we did not find an impact on morbidity or mortality with the use of HES instead of HA. Again, these results are in accord with those of our two prospective studies, although those trials were not sufficiently powered to assess the safety of 6% HES 130/0.4. Our results are also in agreement with those of Sümpelmann *et al*., who performed a before-after study with the aim of assessing the incidence of adverse reactions associated with the use of 6% HES 130/0.42 [28]. In their study, which included 1,130 children undergoing surgery and exposed to 6% HES 130/0.42, they did not report any adverse reactions and observed only non–clinically relevant changes in metabolic parameters (plasma chloride ion concentration, excess base). Recent systematic reviews and meta-analyses have confirmed that the use of 6% HES 130/0.4 is not associated with a deleterious effect on postoperative morbidity and mortality [13,27,29,30], in contrast to what has been observed in critically ill patients [31-33]. Our results are also in line with those of another recent study in which researchers reported a comparable efficacy and safety profile between HA and HES in adult patients undergoing cardiac surgery [34]. Although the requirement for renal replacement therapy is usually infrequent in children undergoing cardiac surgery, the maximal postoperative creatinine level was not increased in children who received HES, and it was significantly lower than in patients who received HA. Although these results should be interpreted in the context of the limitations described below, we do not report any signal of potential harmful effect associated with HES administration in children undergoing cardiac surgery in the present study.

The results of our study should be interpreted with consideration of its limitations. This study was not a randomized controlled trial, and, although powerful adjustment methods were used, the probability of unrecognized confusion bias persists. We performed propensity-matched analysis using 13 variables defined *a priori* as possible factors that could influence the difference between the two study groups. Although the choice of these 13 factors could be extensively discussed, we used the most relevant demographic and clinical parameters that could have influenced the repartition between both study groups.

Aprotinin was withdrawn from the market after the publication of the BART study [35]. We therefore switched from aprotinin to tranexamic acid in 2008, and this might have influenced our results regarding perioperative blood loss. However, according to a recent meta-analysis, there is no prospective study that has directly compared aprotinin to tranexamic acid in pediatric cardiac surgery [36]. In a recent retrospective analysis including more than 22,000 patients, Pasquali *et al*. reported that, compared with aprotinin, tranexamic acid was associated with significantly reduced mortality and bleeding requiring surgical intervention [37]. However, the statistical analysis used for our different outcome parameters was adjusted for the type of antifibrinolytic agent used. We therefore believe that the switch from aprotinin to tranexamic acid in 2008 did not significantly influence our results.

In this study, we voluntarily avoided adjusted analysis with the year of the surgery, because this parameter would covary with the change from HA to 6% HES 130/0.4, which would have biased the results of our regression analyses. Although we performed the propensity-matched analysis on the basis of 13 relevant parameters, we agree that the results we observed cannot be attributed solely to the different colloids administered. The changes in our priming strategy should be considered as part of a multimodal approach aimed at improving blood management strategy in pediatric patients, which includes the adoption of a restrictive transfusion policy.

## Conclusions

The results of this large propensity-matched analysis confirm those obtained in two previous prospective randomized trials. They show that the use of 6% HES 130/0.4 for volume replacement in children undergoing cardiac surgery with CPB was an effective and safe alternative to HA. Use of 6% HES 130/0.4 was associated with a less positive intraoperative fluid balance, which might have an impact on early postoperative fluid overload. Further large studies are needed to assess whether reduction in intraoperative fluid accumulation could have a significant impact on postoperative morbidity and mortality. Owing to the higher cost of HA, 6% HES 130/0.4 can be considered as a safe and cost-effective alternative in pediatric cardiac surgery.

## Key messages

- The use of HES for volume replacement in children undergoing cardiac surgery with CPB appears to be as safe as HA.
- The use of HES might be associated with less fluid accumulation, which might have a significant impact on postoperative morbidity and mortality.

## Abbreviations

ACT: Activated clotting time
ASA: American Society of Anesthesiologists
CPB: Cardiopulmonary bypass
EBV: Estimated blood volume
FFP: Fresh frozen plasma
HA: Human albumin
HES: Hydroxyethyl starch
ICU: Intensive care unit
IN: Total fluid volume administered intraoperatively, including cardiopulmonary bypass prime volume, cardioplegia volume and all fluid administered with drugs and flushes of invasive pressure lines
MUF: Modified ultrafiltration
OUT: Total fluid output, including blood loss, urine output and amount of ultrafiltration
PICU: Pediatric intensive care unit
RBC: Red blood cells
UFH: Unfractionated heparin
WLS: Weighted least squares
